# Electron-ion equilibration in superheated gold

**DOI:** 10.1038/s41467-026-74346-9

**Published:** 2026-06-18

**Authors:** Travis D. Griffin, Dirk O. Gericke, Daniel Haden, Hae Ja Lee, Eric Galtier, Eric Cunningham, Dimitri Khaghani, Michael Larsen, Lennart Wollenweber, Ben Armentrout, Carson Convery, Karen Appel, Gilliss Dyer, Luke B. Fletcher, Sebastian Göde, J. B. Hastings, Jeremy Iratcabal, Emma E. McBride, Jacob Molina, Giulio Monaco, Landon Morrison, Hunter Stramel, Sameen Yunus, Ulf Zastrau, Siegfried H. Glenzer, Gianluca Gregori, Bob Nagler, Thomas G. White

**Affiliations:** 1 Department of Physics, University of Nevada, Reno, Nevada USA; 2 European XFEL, Holzkoppel 4, Schenefeld, Germany; 3 Centre for Fusion, Space and Astrophysics, Department of Physics, University of Warwick, Coventry, UK; 4 SLAC National Accelerator Laboratory, Menlo Park, California USA; 5 Columbia University, New York, New York USA; 6 Queen’s University Belfast, University Rd, Belfast, UK; 7 Department of Astrophysical Sciences, Princeton University, Princeton, New Jersey USA; 8 Princeton Plasma Physics Laboratory, Princeton University, Princeton, New Jersey 08540 USA; 9 Department of Physics and Astronomy “Galileo Galilei”, University of Padova, Via F. Marzolo, 8 - 35131, Padova, PD Italy; 10 University of Oxford, Parks Road, Oxford, UK; 11 Department of Physics, University of California, Merced, California USA

**Keywords:** Laser-produced plasmas, Astrophysical plasmas, Structure of solids and liquids, Phase transitions and critical phenomena

## Abstract

Electron-ion equilibration dynamics in samples driven into superheated states with multi-eV electron temperatures represent a fundamental process in nonequilibrium physics. A clear picture of the evolving ion temperature is essential for understanding the strength of the electron-ion coupling. However, direct, model-independent measurements of the ion temperature in laser-irradiated samples have remained experimentally elusive. Using inelastic X-ray scattering with meV-resolution in gold samples, the ion response to ultrafast heating by the hot electrons can be resolved, quantifying the electron-ion equilibration dynamics. We report measurements revealing a strongly enhanced energy transfer rate compared to that in weakly excited gold. Moreover, we obtain a quasi-constant electron-ion coupling at the highly elevated electron temperatures. These results place new constraints on electron-ion energy transfer in warm dense matter and establish a path toward quantitative benchmarking of models for nonequilibrium dynamics.

## Abstract (web summary)

Direct measurements of ion temperature in superheated gold, using meV-resolution inelastic X-ray scattering, reveal fast electron-ion equilibration dynamics. This model-independent approach quantifies energy transfer rates, providing critical insights into nonequilibrium processes in warm dense matter.

## Introduction

From planetary and stellar physics to the cutting edge of material engineering, ultrafast laser irradiation has emerged as a versatile and powerful tool for investigating materials under extreme conditions. Key applications include inertial fusion energy (IFE)^1,2^, warm dense matter (WDM) studies on basic science and laboratory astrophysics^3–6^, materials science^7–10^, and precision laser machining^11,12^. However, intense laser radiation of solids drives electrons far out of equilibrium, initiating a cascade of relaxation processes. In metallic targets, the interaction initially generates a population of ballistic electrons that thermalize with the conduction electrons within ~500 fs, while the ions remain near room temperature^13^. The subsequent equilibration of the electron and ion temperatures occurs on the picosecond timescale, but the underlying mechanisms remain poorly understood, even for a well-studied material such as gold.

A central obstacle for experimental investigations is the challenge of directly measuring the ion temperature (*T*_*i*_) in laser-heated solids. Inferring data by matching models to indirect observables—such as structural or optical changes in the sample—has led to persistent disagreements among experiments and between experiment and theory^14–19^. Consequently, predictions of the electron-ion coupling parameter (*g*_*ei*_) in gold under WDM conditions differ by up to an order of magnitude, with some studies suggesting a strong dependence on both ion and electron temperatures ^20^. Compounding the challenge, many previous ultrashort laser-pulse studies have assumed that the initial internal energy scales directly with incident fluence^21–24^, an assumption that can introduce uncertainties of up to 50%^7^. Therefore, direct, model-independent measurements of ion temperature, particularly at the high intensities where extreme superheating occurs^25^, are essential for constraining theory and advancing our understanding of nonequilibrium energy transfer in dense plasmas.

Here, we use a meV-resolution inelastic X-ray scattering platform at the LINAC Coherent Light Source to directly measure the evolution of the ion temperature in gold driven by an optical laser at intensities *I* > 10^15^ W/cm^2^, accessing previously unexplored superheated solid states^25^. This model-independent approach, based solely on Doppler broadening of the scattered signal, enables reconstruction of the full temporal equilibration profile. Using this method across four laser fluences, we extract the electron-ion coupling parameter through a Bayesian two-temperature model (TTM) analysis, uncovering substantially enhanced equilibration dynamics compared to room-temperature gold, with saturation observed at the highest intensities.

## Results

### Experimental platform

Recent developments at the MEC end-station at LCLS, providing narrow-bandwidth (50 meV) X-ray pulses^25,26^, now enable the spectral resolution needed for truly model-independent lattice temperature measurements. This platform was initially employed in a forward-scattering configuration to measure collective ion motion, allowing the determination of bulk properties such as sound speed and phonon modes in WDM samples^27–29^. In a back-scattering geometry, the scattering signal becomes sensitive to single-particle motion, directly probing the one-dimensional ion velocity distribution. Our recent work demonstrated that the spectral width of the scattered X-rays can be related to *T*_*i*_ through Doppler broadening

1 $${T}_{i}=\frac{{m}_{i}{c}^{2}}{32\,\,{{\rm{ln}}}\,2}{\left(\frac{\Delta E}{{E}_{0}}\right)}^{2}\,,$$

where *m*_*i*_ is the ion mass, *E*_0_ is the initial X-ray energy, and *ΔE* is the thermal broadening full-width at half-maximum^25^.

**Fig. 1:**
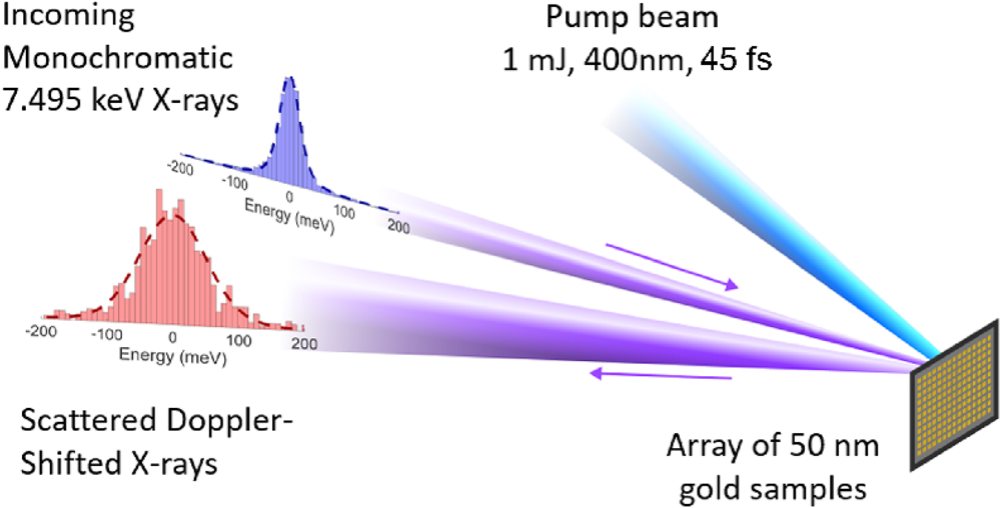
Schematic of the short-pulse laser heating and photon scattering from a monochromated XFEL pulse. Each X-ray pulse has a central energy of *E*_0_ = 7491.9 eV and a very narrow bandwidth of *ΔE*/*E*_0_ = 5 × 10^−6^ (see “Methods”). The 50-nm-thick gold sample is irradiated for 45 fs by a 400 nm wavelength short-pulse laser. The optical laser was focused to a spot approximately 100 *μ*m in radius and calibrated using a camera outside the vacuum chamber. The incoming highly monochromated XFEL radiation (example spectrum is shown in blue) experiences Doppler broadening due to the temperature distribution of the ions (resulting spectrum shown in red). The scattered signal is collected using three Si (533) diced analyzer spectrometers, at a 170^∘^ back scattering angle, and an X-ray camera.

Since the temperature-induced broadening is on the order of 100 meV, achieving this measurement requires the highest possible spectral resolution. This can be achieved by the experimental configuration described in ref. ^25^ and sketched in Fig. 1. Here, we measured the temporal evolution of the ion temperature for four different laser intensities. To construct a time sequence, the delay between the optical heater beam and the X-ray probe beam was varied from −1 to 50 ps, capturing the complete equilibration profile. Multiple shots were acquired at each delay to accumulate sufficient photons for high-quality spectra.

Each spectrum was fitted with a Voigt profile using a maximum likelihood estimator. Around 300 detected photons (~150 shots) were typically required for a reliable fit with acceptable uncertainties. The additional broadening extracted from the fit was converted to temperature using Equation (1), and confidence intervals were obtained by bootstrapping following the approach in ref. ^25^.

### Determining electron-ion coupling parameter

**Fig. 2:**
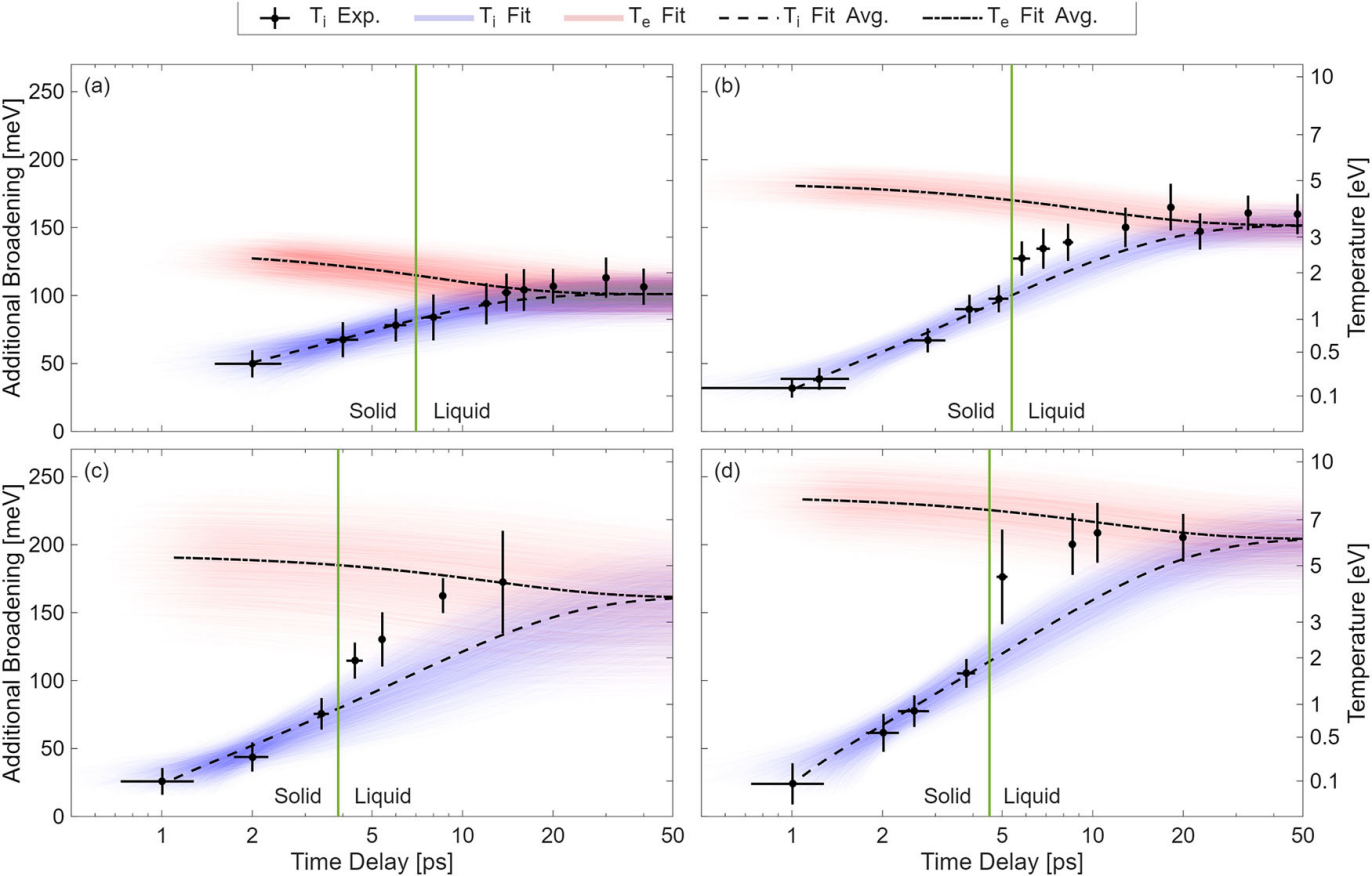
Temperature evolution in strongly driven gold. Panels (**a)**–(**d)** correspond to laser fluences of 1.0 ± 0.25 J/cm^2^, 1.9 ± 0.5 J/cm^2^, 2.0 ± 0.5 J/cm^2^, and 4.9 ± 0.5 J/cm^2^, respectively. Error bars represent the standard time delay separation and the standard deviation of the Gaussian broadening distribution of the scattering signal. Employing a Bayesian model, we fit the temperature profiles with a two-temperature model (see Eqs. (2, 3)) to obtain acceptable limits on the electron-ion coupling parameter, see “Methods” for full details. The red and blue bands represent the posterior distributions for the electron and ion temperatures, respectively, with each panel showing (**a)** 5424, (**b)** 2741, (**c)** 5010, and (**d)** 3996 individual temperature profiles. Before melting, indicated by the vertical green line in each panel, the temperature profiles fit well with a constant *g*_*ei*_. In all but the lowest laser fluence case, the broadening signal exhibits a sharp jump immediately after melting, and the associated temperatures deviate from the extrapolation of the solid-regime fit.

The resulting evolution of the ion temperature for four cases with increasing drive intensity are shown in Fig. 2. Increasing fluence leads to higher final equilibration temperatures and elevated initial electron temperatures. Although the ion temperature rises faster for stronger drives, the relaxation time is similarly increasing, illustrating the need for more detailed modeling to see the trend in the electron-ion energy transfer rate. The electron-ion equilibration dynamics were modeled using a TTM

2 $${c}_{e}\,\frac{d{T}_{e}}{dt}=-{g}_{ei}({T}_{e}-{T}_{i})+S(t=0),$$

3 $${c}_{i}\,\frac{d{T}_{i}}{dt}={g}_{ei}({T}_{e}-{T}_{i}),$$

where *c*_*e*_ and *c*_*i*_ are the electron and ion specific heat capacities taken from ref. ^30^. The term *S*(*t* = 0) represents the initial source term corresponding to the energy density deposited into the system. This term is traditionally used but is excluded from the present analysis due to its inherent errors. Instead, the system is initialized with the lowest ion temperature and the corresponding electron temperature, which is obtained from the total energy of the equilibrated system. This approach circumvents systematic errors in the initial energy density, e.g., from reflection or heat transport, and relies solely on model-independent measurements (see “Methods”).

The measured ion temperatures were fit to the TTM within a Bayesian framework^31^, allowing simultaneous inference of the electron-ion coupling parameter and the initial electron temperature. Posterior likelihood distributions provided uncertainties on these parameters, shown as the shaded regions in Fig. 2 (see “Methods” for the Bayesian analysis). The solid-liquid phase transition, indicated by the vertical green lines in Fig. 2, was determined from the simultaneous X-ray diffraction measurements (see “Methods”), which showed a clear loss of the (111) and (200) crystal peaks upon melting. Across all cases, the data in the solid regime are consistent with a constant electron-ion coupling parameter, even at the highest fluences producing extreme superheating, implying minimal dependence on lattice temperature in this temperature range.

## Discussion

In all but the lowest-fluence data set, the TTM results, using the electron-ion coupling parameter derived from solid-phase temperature evolution, deviate from the experimental data after melting. Beyond this point, the spectral broadening and ion temperature increase considerably in a fluence-dependent manner, with the greatest jump observed at the highest fluence. We can identify three possible sources for this behavior: (1) an increase in the electron-ion coupling parameter in the liquid phase, (2) internal energy differences between the solid and liquid phases, and (3) hydrodynamic expansion. While a combination of these effects cannot be ruled out, disentangling them is beyond the scope of this study. Consequently, we restrict our analysis to the solid phase, where the density remains constant, as confirmed by the stationary Bragg peak position in the XRD signal^25^. This conclusion is further supported by frequency-domain interferometry measurements^14,16^, which indicate no expansion prior to melting and, even after melting, the expansion velocity remains less than 10% of the ion thermal velocity.

**Fig. 3:**
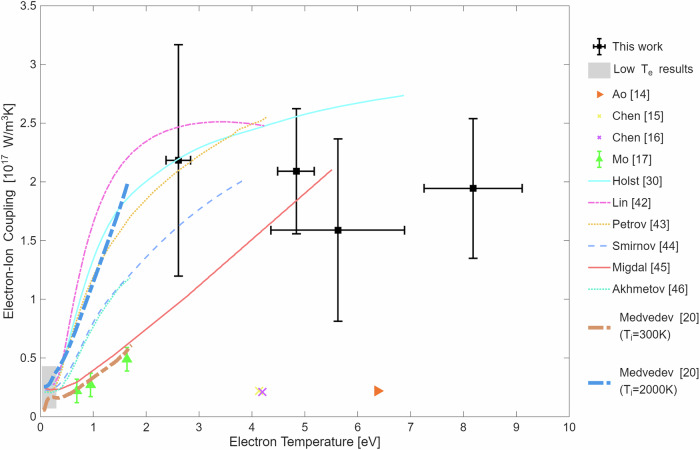
Average electron-ion coupling values extracted from the Bayesian posterior distributions with initial electron temperatures. Initial electron temperatures were determined from the model-independent ion temperature measurements produced with a Bayesian implementation of a two-temperature model. Error bars represent a 1*σ* standard deviation of the respective mean value. Relative to other measurements at comparable electron temperatures^14–17^, strong electron-ion coupling is observed for all conditions studied. Several theoretical predictions align qualitatively in this regime without completely reproducing the experimental behavior^20,30,42–46^. The gray band denotes previously reported experimental values obtained in the low-excitation regime (i.e., low initial energy density)^21,22,41,47–54^.

We compare our results with theoretical predictions of the electron-ion coupling parameter and prior experiments in Fig. 3. Across the range of 2–9 eV electron temperatures, our measurements exceed the near-room-temperature gold results by about an order of magnitude. Furthermore, our data show little or no increase with electron temperature. This plateau contrasts with most models—within the region where their curves overlap our data, which generally predict a monotonic rise. Our lowest-fluence point carries large uncertainties (limited photon statistics and a shallow fitted temperature slope) but remains consistent with this trend. At higher electron temperatures, few theoretical predictions have been reported, limiting direct comparison. The observed plateau may be explained by the dynamics in Pauli blocking, which prevents *d*-electrons in cold gold from scattering. At elevated electron temperatures, promotion of *d*-electrons to the conduction band creates *d*-band holes, permitting greater *d*-electron participation in energy exchange with the ions^32^. When full *d*-electron participation is reached, the effect saturates and the energy exchange rate plateaus at a quasi-constant elevated level.

Relative to previous ultrafast measurements on gold^14–19^, we observe substantially higher electron-ion coupling. This is consistent with recent observations in copper, which show strongly enhanced equilibration rates for hotter systems relative to ambient conditions^23,24,33–36^. By contrast, earlier studies on gold used lower energy inputs, producing smaller temperature differences. Furthermore, they used structural or optical proxies to infer electron and ion temperatures. Here, we probe a highly nonequilibrium regime characterized by high electron and exceptionally high ion temperatures, directly measured via meV-resolution inelastic X-ray scattering. The few models incorporating ion temperature dependence (e.g., Medvedev et al.^20^) predict enhanced coupling at higher ion temperatures, consistent with our results. Therefore, our findings indicate increased coupling in strongly superheated noble metals, motivating models that incorporate ion temperature dependence and saturation at high electron energy density.

The findings are highly relevant to inertial confinement fusion and inertial fusion energy schemes. The nonequilibrium dynamics of these superheated samples are directly relevant to such setups^1^. Our data serve as a valuable benchmark for predictive modeling, as the behavior observed is not yet fully captured by any theory. Future experiments in this regime could refine the role of extreme superheating and, in particular, extend the investigation of the electron-ion coupling parameter beyond the phase boundary into the fluid regime, where considerable uncertainties remain as well^37,38^.

## Methods

### X-ray probe characterization

**Fig. 4:**
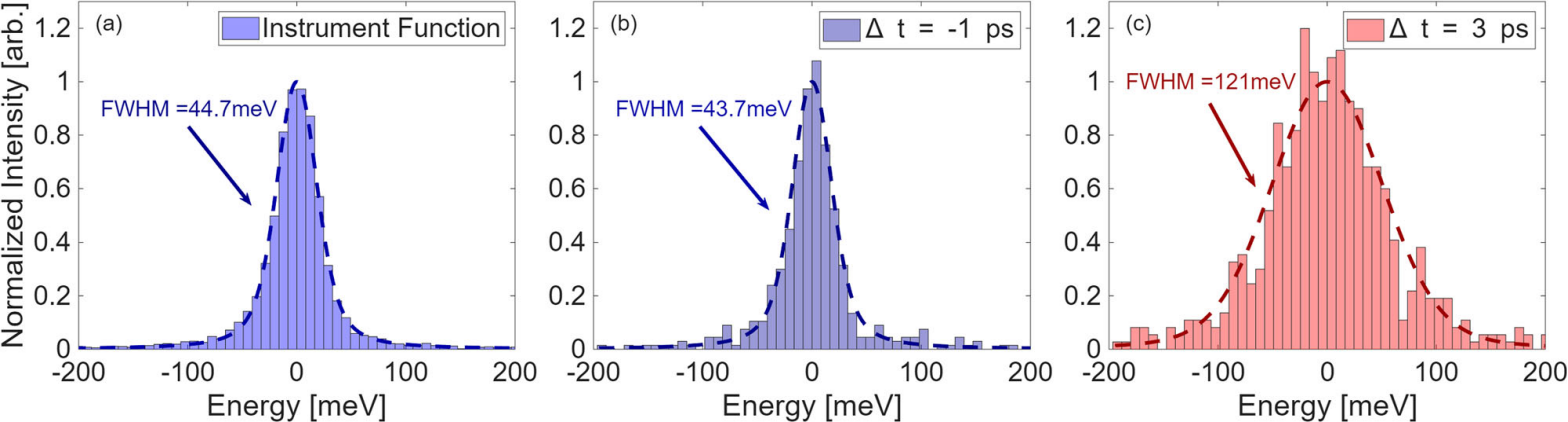
Inelastic X-ray scattering spectra for 50 nm gold samples. Individual panels showing (**a**) the instrument function (X-ray only), (**b**) a spectrum at −1 ps delay between the X-ray probe and optical pump, and (**c**) the X-ray probe delayed by 3 ps after optical pump, exhibiting a significantly broader spectrum. Voigt profile fits to the spectra enable ion-temperature determination using Eq. (1).

The experiment was conducted using the X-ray free-electron laser probe at the Matter in Extreme Conditions (MEC) end station of the LINAC Coherent Light Source (LCLS) beamline at SLAC National Laboratory. The X-ray beam was tuned to  ~ 7.49 keV with a nominal pulse energy of  ~ 100 *μ*J and a pulse duration of 50 fs, operating in self-seeding mode with a bandwidth of approximately 1 eV. The beam was passed through a four-bounce silicon channel-cut (533) monochromator at a Bragg angle of 87.7^∘^, reducing the bandwidth to a nominal  ~ 30 meV, and was focused onto the target to a 20 *μ*m diameter spot. A diced crystal analyzer was positioned at a scattering angle of 170^∘^, corresponding to a scattering vector of 7.6 Å^−1^. The spectrometer employed an energy-dispersive Johann geometry based on a Rowland circle with a diameter of 1 m, with spectra recorded on an ePIX100 detector with 50 *μ*m pixels. The combined energy resolution of the monochromator and analyzer was ~50 meV, as shown by the instrument function in Fig. 4(a). Subsequent temperature spectra are accumulated for each time delay between the optical laser and X-ray probe. Spectra corresponding to −1 ps and 3 ps are shown in Fig. 4(b), (c), respectively.

### Determination of melting

**Fig. 5:**
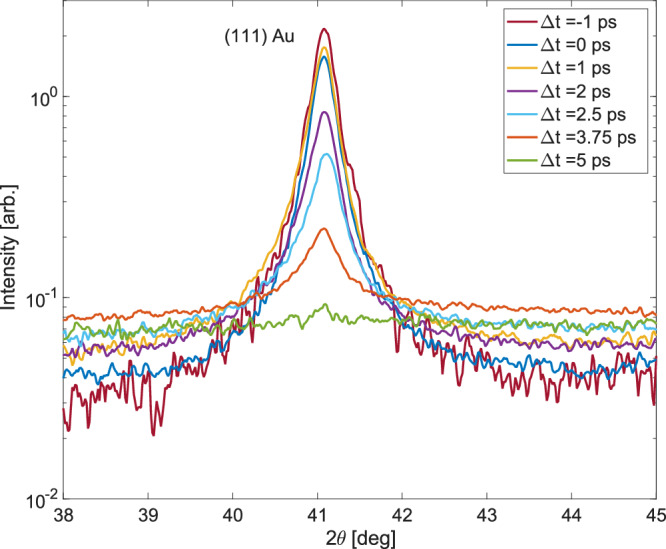
Evolution of the gold (111) Bragg peak diffraction. For the case with the highest fluence, including data acquired prior to melting as well as the first time step after melting. The solid-to-liquid phase transition is identified by the complete disappearance of the (111) diffraction peak and is indicated by a vertical green line in Fig. 2(d).

We utilize XRD to determine the melt time of each sample. In the homogeneous melting regime^17^, the loss of the (111) diffraction peak of solid gold coincides with the onset of melting. For each ion-temperature measurement, simultaneous XRD data were collected. An example for the highest fluence case is shown in Fig. 5. The peak position, which remains constant prior to melting, is used to constrain the sample density in the pre-melt state. The melt time, indicated by a green vertical line in Fig. 2(d), is defined as the midpoint between the last time step containing a distinguishable solid (111) diffraction peak and the first measurement corresponding to a fully liquid state lacking this peak.

### Bayesian parameter estimation

We quantify uncertainties by fully Bayesian inference in a model that couples the two-temperature dynamics to the measurement process and samples the joint posterior of the experiment start time *t*_0_, the initial temperatures (*T*_*e*_(*t*_0_), *T*_*i*_(*t*_0_)), and the electron-ion coupling parameter *g*_*ei*_^31^. We place a Normal prior on *t*_0_ centered at the first timestamp; Normal priors on *T*_*e*_(*t*_0_) and *T*_*i*_(*t*_0_) centered at their respective first measurements; and a weakly informative Uniform prior on *g*_*ei*_. We assume independent Gaussian errors on the measured ion temperatures, which we combine with a weighted least squares sum. Per-point timing jitter is handled by evaluating the model at (*t* − *σ*_*t*_, *t*, *t* + *σ*_*t*_) and aggregating the three Normal terms with fixed, normalized weights. We sample the posterior with a robust adaptive Metropolis-Hastings kernel until convergence and report posterior summaries for *g* as the posterior mean and standard deviation. All inference was performed in *Julia*, using Turing.jl (with the RAM kernel from AdvancedMH.jl) for sampling and DifferentialEquations.jl for solving the two-temperature ODEs.

### Determination of initial electron temperature

Deposited energy density, *ϵ*, was determined from late-time (> 9 ps) ion temperatures measured after full electron-ion equilibration. The initial electron temperature is obtained by inverting the equation of state via

4 $$\epsilon ({T}_{{{\rm{final}}}})=\int\limits^{{T}_{e}^{{{\rm{init.}}}}}_{300\,{{\rm{K}}}}{c}_{e}(T)\,dT+\int\limits^{{T}_{i}^{{{\rm{init.}}}}}_{300\,{{\rm{K}}}}{c}_{i}(T)\,dT\,,$$

as before, *c*_*e*_ and *c*_*i*_ are the specific heat capacities of the electrons and ions, respectively. Values for the heat capacities were taken from Holst et al.^30^, and the internal energy was computed with the Frankfurt equation of state (FEOS)^39^. Thus, the initial electron temperature and energy density can be written as a function of the late-time ion temperature measurement, which we obtain via inelastic X-ray scattering.

As the density of the samples cannot be constrained after the melting experimentally, late-time temperature measurements may be systematically effected by hydrodynamic expansion. Decoupling temperature and expansion is enabled by results from FDI measurements at comparable conditions, which suggest that after melting, the surface expansion velocity is significantly lower than the thermal velocity and accounts for less than 10% of additional broadening^14,16^. As the directed velocity component decreases further deeper into the bulk of the sample, its contribution to temperature estimation is likely well below 10%. Therefore, any observed discontinuity in the temperature spectrum is primarily attributable to a real jump in temperature at late time.

### Reporting summary

Further information on research design is available in the Nature Portfolio Reporting Summary linked to this article.

## Supplementary information

Reporting Summary

Transparent Peer Review file

## Data Availability

The datasets generated during and/or analysed during the current study are available in the Zenodo repository, 10.5281/zenodo.19438098^40^.

## Code availability

The codes used for the analysis described in the manuscript are available from the corresponding authors upon request.
