# Supplementary material for: Electron-ion equilibration in superheated gold
Source: Nat Commun. 2026 Jun 18;17:7567. doi: 10.1038/s41467-026-74346-9 (PMC13415882; doi:10.1038/s41467-026-74346-9)
Supplement: Supplementary file 2 — Transparent Peer Review file [file 41467_2026_74346_MOESM2_ESM.pdf]

Peer Review File

Electron-Ion Equilibration in Superheated Gold

Corresponding Author: Dr Travis Griffin

This file contains all reviewer reports in order by version, followed by all author rebuttals in order by version.

Version 0:

Reviewer comments:

Reviewer #1

(Remarks to the Author)
Superheating is a very interesting subject area and this paper connects to time dependent warm dense matter, which is very topical. Exploration of ultra-fast processes in challenging experiments like this promise to uncover a great deal of important information that can help modelling. It is true, as stated by the authors, that there have been widely different values for electron-ion coupling constants in the literature and more experimental data of this quality is needed.

I have some questions about the manuscript.

First, can I clarify how melting is determined? Is this from observing the diffraction change to a liquid like structure factor? This is what is suggested on page 6 but I was not sure.

Is the illustration in figure 1 an actual spectrum taken or a schematic?

Page 4 typo "Although the ion temperature rise faster for...", should be "Although the ion temperature rises faster for.."

Says 45 fs in caption of figure 1 but 40fs in figure.

On the whole I think this is a useful paper and can be published with some minor corrections as above, the typos and maybe clarifying (just a little more) how the melting line is determined for figure 2.

Reviewer #2

(Remarks to the Author)
The work utilizes a recently developed capability at the MEC station at the LCLS X-ray FEL to measure the ion temperature in a strongly driven sample through the observed Doppler broadening in a high resolution inelastic scattering measurement.

Based on this ability to reliably measure the ion temperature following laser excitation, the electron and ion temperatures in gold foils are followed over a time period between -1 and 50 ps after excitation by a fs laser pulse at intensities higher than 10^15 W/cm2.

As a central result, the electron-ion coupling parameter (g_ei) is obtained from the analysis. It is shown that this parameter is substantially higher than previously reported in gold. Before melting, when the gold is still solid, but superheated, the temperature profiles fit well with a constant g_ie for all measured pump laser fluences. In summary, the g_ie appears to be significantly higher than previously reported and displays a plateau in the electron temperature range between 2 and 8 eV.

It is also reported that, for all but the lowest laser fluence case, the measured ion temperatures exceed those predicted by the TTM in the liquid phase. Possible reasons for this are briefly hypothesized but this interesting result is not investigated in any detail.

In my opinion, the characterization of materials driven far out of equilibrium is of high interest based on its importance for the areas of application outlined in the introduction of the manuscript.
I believe the methodology of the study is sound with improvements based mainly on the development of the new capability to measure the ion temperature. The present work does not represent a huge leap from the recent publication from (partly)

the same authors (https://doi.org/10.1038/s41586-025-09253-y). But, in my opinion, it represents a sufficient development to warrant publication in Nature Communications. I find that the presented work supports the conclusions made and that it is presented with sufficient detail to allow reproduction. For these reasons I recommend the work for publications in Nature Communications after a few points have been addressed.

I do not find information about the pulse duration and intensity on the sample for the X-ray pulses used for the inelastic scattering measurement that determines the ion temperature. This should be added, together with a discussion (or reference) that verifies that there is no risk of X-ray light/matter interactions occurring during the X-ray pulse having any effect on the scattering experiment. That the effects observed in the scattering experiment to determine the ion temperature are solely from the pump pulse and does not have any contribution from effects initiated by the first part of the X-ray pulse.

In a wide range of X-ray FEL experiments, from the very first LCLS experiments [doi:10.1038/nature09177] until present day [https://doi.org/10.1107/S2052252525002660] it has been an active area of research to investigate how, at high intensities and depending on the pulse duration, processes in the sample, during the pulse, affect the outcome of the measurement. I am not aware if this has been studied in detail for the Doppler shift used to determine the ion temperature but as this is a common issue in X-ray FEL experiments I think it should be addressed. In particular as I expect that the X-ray pulse duration is somewhat longer than in many other X-ray FEL diffraction/scattering experiments.

On line 121, p6 it says “the observed trend..” Clarify what is meant by “trend”. Are you referring to the plateau in g_ei ?

A few details:
On line 112 “FDI” measurements are mentioned. What is FDI an abbreviation for ?
On line 111 and 112 where it is written that “Density remains constant … confirmed by Bragg diffraction”. I think a reference would be appropriate. I suppose it is ref. 24.
In fig. 3. Check correspondence between the reference numbers in the figure and the actual numbers in the list of references. I think there are discrepancies. E.g. in fig 3, it says: “Ao[11]”, “Chen [12]” and “Chen [14]” but in the list of references Ao is [12] and Chen is [13] and [15].

Reviewer #3

(Remarks to the Author)
This manuscript by Griffin et al presents experimental measurements of ion temperatures in super heated gold obtained through inelastic x-ray scattering measurements. These ion temperatures are then used to model the electron ion relaxation dynamics, with the main goal to extract the electron-ion coupling parameter. Their main result is that they explore a region of temperature where scarce data and calculations exist, and that the apparent absence of variation of the coupling parameter is calling into questions current models.

Based on an outsider view, I believe that these results, if they can be established convincingly, can represent an important advance in the specialized field of dynamics of plasmas relevant to astrophysical or inertial fusion research. I find however that in its current presentation, the manuscript fails at establishing why a more general audience should be interested in these results. Perhaps a better description of the motivations behind this work, and how this kind of studies could provide essential building blocks towards important developments in the relevant scientific fields would help.

On the other hand, at this time I also find the manuscript lacking as far as presenting convincing results. Some of this view stems from the general lack of details that I was expecting to be presented in a supplementary file. For example, More details about the experimental setup, some direct demonstration of the measured x-ray spectra, including some measured with no laser, and some with negative delays to better be able to establish how convincing this data set can be.

Figure 2 presents the main experimental results, and there are several potential red flags here.
1- In each case a line separating solid and liquid phase is presented. Where does that come from? As it is here, the reader has no idea how this is established.
2- Panel b and c are said to present data with very similar average laser fluence and yet look quite different. I have to say that this does not provide much confidence in the ability to precisely determine the laser parameters.
3- The text explains that while the ion temperature is directly extracted from the bandwidth broadening, the electron temperature is derived from the total energy of the equilibrated system. In Methods we read that this is extracted from a late time ion temperature. Are there more data than presented in fig2 for late times? If about 50ps is late time, panel 3c is definitely lacking, and one could imagine a significantly different fit.

Fig 3 shows the coupling parameter as a function of electron temperature. It is not very clear how these 4 data points are constructed. They should not exactly correspond to the 4 laser fluences, and if one looks at all the data points in the solid regions, there should be more than 4 extracted points. It seems that there would be quite a dependence on the understanding of the late time behavior, since the electron temperature model is linked to it. In that regard, the fact that the text explains that the behavior in the melted region is not understood in most cases looks like a big concern. In any case, a much better description of how this graph is obtained, and especially the error bars, is needed.

Overall, I found this manuscript frustrating in its presentation. At this time, I can only recommend major revisions to help make a more convincing case for the accuracy of the results, and their general importance.

Version 1:

Reviewer comments:

Reviewer #1

(Remarks to the Author)
The new manuscript has addressed the issues and is ready for publication.

Reviewer #2

(Remarks to the Author)
My concerns have been addressed in the revisions. I also appreciate the clarifications offered by the changes and additions made in response to the points raised by the other reviewers. My opinion is that the manuscript can be accepted.

Reviewer #3

(Remarks to the Author)
The resubmission by the authors carefully considers all three reviewers comments. While I had recognized that the results could be of high interest for the specialized community, the reworked introduction does present the work in larger context. My issues with having too many specialized aspects of the experiment and analysis assumed to be known or needing to refer to previous papers without knowing if conditions may have changed, have been alleviated by some much needed additions to the Method section.
The additional explanations about how the electron temperature is extracted, about the liquid phase and about experimental measurements of the scattered spectrum improve the presentation and help convince the reader of the validity of the results.

I now recommend publication in Nature communications.

Open Access This Peer Review File is licensed under a Creative Commons Attribution 4.0 International License, which permits use, sharing, adaptation, distribution and reproduction in any medium or format, as long as you give appropriate credit to the original author(s) and the source, provide a link to the Creative Commons license, and indicate if changes were made.
In cases where reviewers are anonymous, credit should be given to 'Anonymous Referee' and the source.
The images or other third party material in this Peer Review File are included in the article's Creative Commons license, unless indicated otherwise in a credit line to the material. If material is not included in the article's Creative Commons license and your intended use is not permitted by statutory regulation or exceeds the permitted use, you will need to obtain permission directly from the copyright holder.
To view a copy of this license, visit https://creativecommons.org/licenses/by/4.0/

Dear Reviewers,

We greatly appreciate the insights and feedback that you have provided, the constructive criticism in your comments has improved the manuscript immensely. Below we have provided individual responses to all comments, questions, and concerns that you have raised. Our responses are highlighted in blue below. Modifications to the original manuscript and Methods sections are highlighted in red. We also corrected a labeling error on the right-hand y-axis of Figure 2. This correction does not affect the data, analysis, or conclusion of the manuscript. We look forward to your continued feedback and are grateful for your contributions to this work.

Response to Referee 1

Superheating is a very interesting subject area and this paper connects to time dependent warm dense matter, which is very topical. Exploration of ultra-fast processes in challenging experiments like this promise to uncover a great deal of important information that can help modelling. It is true, as stated by the authors, that there have been widely different values for electron-ion coupling constants in the literature and more experimental data of this quality is needed.

I have some questions about the manuscript.

First, can I clarify how melting is determined? Is this from observing the diffraction change to a liquid like structure factor? This is what is suggested on page 6 but I was not sure.

We used the disappearance of solid diffraction lines to identify the melt, which we discussed in our previous publication [1]. However, as all three reviewers have asked for clarification on this topic, we believe it is worth also including the details in this manuscript. As such, we have added a section in the Methods to fully explain the determination of melting using XRD techniques.

[1] White, T.G., Griffin, T.D., Haden, D. *et al.* Superheating gold beyond the predicted entropy catastrophe threshold. *Nature* **643**, 950–954 (2025). https://doi.org/10.1038/s41586-025-09253-y

Is the illustration in figure 1 an actual spectrum taken or a schematic?

The illustration is an actual spectrum, and we have added clarifying statements in the figure 1 caption. We have also presented the spectra more clearly in the Methods.

Page 4 typo "Although the ion temperature rise faster for...", should be "Although the ion temperature rises faster for.."

Thank you for catching this typo, this has been corrected in the text.

Says 45 fs in caption of figure 1 but 40fs in figure.

This typo in Figure 1 has also been corrected. The correct pulse duration was 45 fs, and is now stated correctly in the Figure.

On the whole I think this is a useful paper and can be published with some minor corrections as above, the typos and maybe clarifying (just a little more) how the melting line is determined for figure 2.

<u>Response to Referee 2</u>

The work utilizes a recently developed capability at the MEC station at the LCLS X-ray FEL to measure the ion temperature in a strongly driven sample through the observed Doppler broadening in a high resolution inelastic scattering measurement.

Based on this ability to reliably measure the ion temperature following laser excitation, the electron and ion temperatures in gold foils are followed over a time period between -1 and 50 ps after excitation by a fs laser pulse at intensities higher than 10^15 W/cm2.

As a central result, the electron-ion coupling parameter (g_ei) is obtained from the analysis. It is shown that this parameter is substantially higher than previously reported in gold. Before melting, when the gold is still solid, but superheated, the temperature profiles fit well with a constant g_ie for all measured pump laser fluences. In summary, the g_ie appears to be significantly higher than previously reported and displays a plateau in the electron temperature range between 2 and 8 eV.

It is also reported that, for all but the lowest laser fluence case, the measured ion temperatures exceed those predicted by the TTM in the liquid phase. Possible reasons for this are briefly hypothesized but this interesting result is not investigated in any detail.

In my opinion, the characterization of materials driven far out of equilibrium is of high interest based on its importance for the areas of application outlined in the introduction of the manuscript.

I believe the methodology of the study is sound with improvements based mainly on the development of the new capability to measure the ion temperature. The present work does not represent a huge leap from the recent publication from (partly) the same authors (https://doi.org/10.1038/s41586-025-09253-y). But, in my opinion, it represents a sufficient development to warrant publication in Nature Communications. I find that the presented work supports the conclusions made and that it is presented with sufficient detail to allow reproduction. For these reasons I recommend the work for publications in Nature Communications after a few points have been addressed.

I do not find information about the pulse duration and intensity on the sample for the X-ray pulses used for the inelastic scattering measurement that determines the ion temperature.

We have added all of the details to the Methods section of the manuscript.

This should be added, together with a discussion (or reference) that verifies that there is no risk of X-ray light/matter interactions occurring during the X-ray pulse having any effect on the scattering experiment. That the effects observed in the scattering experiment to determine the ion temperature are solely from the pump pulse and does not have any contribution from effects initiated by the first part of the X-ray pulse. In a wide range of X-ray FEL experiments, from the very first LCLS experiments [doi:10.1038/nature09177] until present day [https://doi.org/10.1107/S2052252525002660] it has been an active area of research to investigate how, at high intensities and depending on the pulse duration, processes in the sample, during the pulse, affect the outcome of the measurement. I am not aware if this has been studied in detail for the Doppler shift used to determine the ion temperature but as this is a common issue in X-ray FEL experiments I think it should be addressed. In particular as I expect that the X-ray pulse duration is somewhat longer than in many other X-ray FEL diffraction/scattering experiments.

First, and most fundamentally, the relevant timescale for energy coupling from the electrons to the ions is on the order of picoseconds, whereas the X-ray pulse duration is ~50 fs. Since the X-rays primarily couple energy into electrons and our scattering signal is sensitive to ionic features, any X-ray-induced modification of the ion distribution occurs on a timescale that far exceeds the X-ray probe window. Therefore, the X-ray beam cannot influence the ionic scattering feature we measure. Second, by the time the beam reaches the target, it has passed through the monochromator and beam optics, which dramatically reduces the photon flux. This is directly evidenced by our ability to operate continuously at 120 Hz with no discernible change in the scattering signal from the cold target. As a result, a single X-ray pulse eventually brings the sample well below the melt temperature, making any contribution negligible.

On line 121, p6 it says "the observed trend.." Clarify what is meant by "trend". Are you referring to the plateau in g_ei ?

Correct, the word 'trend' on that line was used to indicate the plateau in g_ei. We have changed the word 'trend' to 'plateau' to make this clear.

A few details:
On line 112 "FDI" measurements are mentioned. What is FDI an abbreviation for ?

FDI is an abbreviation for Frequency Domain Interferometry. We have clarified the acronym in the text.

On line 111 and 112 where it is written that "Density remains constant ... confirmed by Bragg diffraction". I think a reference would be appropriate. I suppose it is ref. 24.

Yes, the details are provided in ref. 24 (now 26) and we have added a citation. Additionally, we have added a section in Methods describing this in a little more detail.

In fig. 3. Check correspondence between the reference numbers in the figure and the actual numbers in the list of references. I think there are discrepancies. E.g. in fig 3, it says: "Ao[11]", "Chen [12]" and "Chen [14]" but in the list of references Ao is [12] and Chen is [13] and [15].

Thank you for catching this error. The citations in Figure 3 have been corrected.

<u>Response to Referee</u> <u>3</u>

This manuscript by Griffin et al presents experimental measurements of ion temperatures in super heated gold obtained through inelastic x-ray scattering measurements. These ion temperatures are then used to model the electron ion relaxation dynamics, with the main goal to extract the electron-ion coupling parameter. Their main result is that they explore a region of temperature where scarce data and calculations exist, and that the apparent absence of variation of the coupling parameter is calling into questions current models.

Based on an outsider view, I believe that these results, if they can be established convincingly, can represent an important advance in the specialized field of dynamics of plasmas relevant to astrophysical or inertial fusion research. I find however that in its current presentation, the manuscript fails at establishing why a more general audience should be interested in these results. Perhaps a better description of the motivations behind this work, and how this kind of studies could provide essential building blocks towards important developments in the relevant scientific fields would help.

We would like to reference the responses from Referees 1 and 2 as evidence of the appeal of our work. However, we agree that the introduction did not effectively highlight its broader relevance. To address this, we have added several references to better articulate the general significance of our research, along with additional citations supporting our motivation for submitting to *Nature Communications*.

As outlined in the revised manuscript, our study of ultrafast laser heating of metallic samples is directly relevant to several active research areas, including inertial fusion energy, warm dense matter physics, laboratory astrophysics, materials science, and advanced laser processing. We have also included several review articles that highlight the importance of electron-ion equilibration in these fields.

Furthermore, the newly cited literature, much of which are published in *Nature*-affiliated journals, demonstrates sustained and widespread interest in these topics across high-impact, general-interest venues. Collectively, these works have received substantial citation attention, underscoring both the timeliness of the subject matter and its relevance to a broad scientific readership.

We acknowledge that our study focuses on a single parameter that is important in the modelling of these conditions. However, particularly in metals, the electron-ion coupling parameter remains poorly constrained. As shown in Figure 3, existing model predictions for this parameter vary widely and have yet to reach consensus. Moreover, previous experimental efforts have not provided sufficient benchmarking to reliably inform these models. In the revised manuscript, we have further emphasized this point.

We hope that these revisions clarify the interdisciplinary significance of our study and its suitability for a broad, general-interest journal.

On the other hand, at this time I also find the manuscript lacking as far as presenting convincing results. Some of this view stems from the general lack of details that I was expecting to be presented in a supplementary file. For example, More details about the experimental setup, some direct demonstration of the measured x-ray spectra, including some measured with no laser, and some with negative delays to better be able to establish how convincing this data set can be.

You are correct; we relied too heavily on information presented in our previous manuscript, and we agree that including this information and the figures you suggest would enhance this manuscript. To address this, we have added additional details to the Methods section, including information on the X-ray-only and driven spectra at negative and later times, as well as further insights on X-ray diagnostics.

Figure 2 presents the main experimental results, and there are several potential red flags here.
1- In each case a line separating solid and liquid phase is presented. Where does that come from? As it is here, the reader has no idea how this is established.

We agree that the explanation of the vertical line in each panel of Figure 2 was insufficient. Therefore, we have now explicitly stated in the text and figure caption the origins of this line. Additionally, we have added a section in Methods that fully explains how the melting point was determined using Bragg peak XRD. While the majority of this discussion was included in a previous publication [1], all three reviewers have requested clarification on this topic. Consequently, we have added a section in the Methods to comprehensively detail the determination of melting using X-ray diffraction techniques.

[1] White, T.G., Griffin, T.D., Haden, D. *et al.* Superheating gold beyond the predicted entropy catastrophe threshold. *Nature* **643**, 950–954 (2025). https://doi.org/10.1038/s41586-025-09253-y

2- Panel b and c are said to present data with very similar average laser fluence and yet look quite different. I have to say that this does not provide much confidence in the ability to precisely determine the laser parameters.

The data in Figure 2. panels b) and c) are quite similar, and we did consider combining them into a single spectrum for publication. However, since the two datasets were collected on different target frames and at different times, we felt it would be misleading to combine these two spectra. We also considered excluding panel c) entirely, as its data quality and time extent are not on the same level as the three remaining profiles. Ultimately, we decided to include all data collected during the experiment for transparency and completeness. We note that the final equilibration values from panels b) and c), presented in Figure 4., agree with each other within their respective uncertainties. This indicates self-consistency for all data presented and provides maximum transparency regarding the experimental details.

3- The text explains that while the ion temperature is directly extracted from the bandwidth broadening, the electron temperature is derived from the total energy of the equilibrated system. In Methods we read that this is extracted from a late time ion temperature. Are there more data than presented in fig2 for late times? If about 50ps is late time, panel 3c is definitely lacking, and one could imagine a significantly different fit.

We have defined “late time” to be greater than 9 ps, which is now specified in the corresponding Methods section. This cutoff is used to include all data points that visually indicate an equilibrated ion temperature profile. As you correctly point out, panel c) lacks late-time data, and there is a range of significantly different fits that are consistent with our results. These fits, shown in red and blue in each of the panels, were obtained using Bayesian analysis. These fits are used to calculate the corresponding uncertainty in the initial electron temperature, which is extremely large for this data point, precisely due to the lack of late time data. However, this is still a valid point, it just has a large uncertainty.

Fig 3 shows the coupling parameter as a function of electron temperature. It is not very clear how these 4 data points are constructed. They should not exactly correspond to the 4 laser fluences, and if one looks at all the data points in the solid regions, there should be more than 4 extracted points. It seems that there would be quite a dependence on the understanding of the late time behavior, since the electron temperature model is linked to it. In that regard, the fact that the text explains that the behavior in the melted region is not understood in most cases looks like a big concern. In any case, a much better description of how this graph is obtained, and especially the error bars, is needed.

The initial electron temperatures shown in Figure 3 are determined from the posterior distributions of the two-temperature model (TTM) Bayesian analysis. Importantly, each iteration of the TTM is fitted using a single, constant electron-ion coupling parameter throughout the solid phase, as stated in the manuscript. We find that it is not possible to reliably differentiate multiple electron-ion coupling parameters within the solid-phase data; within the resolution of our uncertainty, an ion-temperature-dependent electron-ion coupling parameter cannot be established. Therefore, a single value is extracted for each fluence case, corresponding to the four points shown in Figure 3.

While we have identified three possible mechanisms for the abrupt change in equilibration upon melting, only rapid expansion would lead to an incorrect determination of the ion-temperature. In contrast, a change in electron-ion coupling or internal energy differences between the liquid and the solid phases would both produce a genuine increase in temperature that is observable in our measurements. Thus, estimates of target expansion at late times can be used to quantify potential errors in the late-time temperature.

Results from Frequency Domain Interferometry (FDI) measurements on comparable targets irradiated with similar fluences [2,3] suggest that the surface expansion velocity is significantly lower than the thermal velocity and accounts for less than 10% of the additional broadening. Furthermore, because expansion velocity decreases deeper into the bulk of the sample, its contribution to temperature overestimation is likely well below 10%. Therefore, in the cases where a discontinuity in the measured temperature is observed, we attribute it primarily to a real increase in temperature upon melting.

We agree that this discussion is essential for the justifying our measurements, particularly in the determination of the total energy of the system. Accordingly, we have incorporated this material in the Methods section, where we describe the determination of the initial state and the extraction of the electron temperatures from late-time ion-temperature measurements.

[2] Ao T. *et al.* Phys. Rev. Lett. **96**, 055001 (2006)

[3] Chen Z. *et al.* Phys. Rev. Lett. **121**, 075002 (2018)

Overall, I found this manuscript frustrating in its presentation. At this time, I can only recommend major revisions to help make a more convincing case for the accuracy of the results, and their general importance.
